# Benzothiazolines Acting as Carbanion and Radical Transfer Reagents in Carbon–Carbon Bond Construction

**DOI:** 10.3390/molecules30081711

**Published:** 2025-04-11

**Authors:** Xiaotang Chen, Bao-Chen Qian

**Affiliations:** College of Medical Engineering, Jining Medical University, Jining 272000, China

**Keywords:** benzothiazolines, carbanion transfer reagents, radical transfer reagents, carbon–carbon bond construction

## Abstract

Traditionally employed as hydrogenation reagents, benzothiazolines have emerged as versatile carbanion and radical transfer reagents, playing a vital role in the construction of various carbon–carbon bonds. The cutting-edge progress in photochemistry and radical chemistry have prompted the study of visible light-driven radical reactions, bringing benzothiazolines into a vibrant focus. Their chemical processes have been uncovered to encompass a variety of activation mechanisms, with five distinct modes having been identified. This work reviews the innovative applications of benzothiazolines as donors of alkyl or acyl groups, achieving hydroalkylation or hydroacylation and alkyl or acyl substitution. By examining their diverse activation mechanisms, this review highlights the potential of benzothiazolines serving as alkyl and acyl groups for further research and development. Moreover, this review will offer exemplary applications and inspiration to synthetic chemists, contributing to the ongoing evolution of benzothiazolines utility in organic synthesis.

## 1. Introduction

**Scheme 1:**
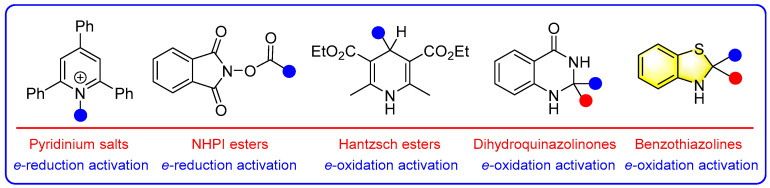
Emerging important radical transfer reagents in radical reactions.

The construction of carbon–carbon (C-C) bonds is one of the fundamental research focuses [1,2,3,4,5]. Generally, the formation of C-C bonds could be achieved through the classical ionic or radical pathways [6,7,8,9,10]. However, with advancements in photochemical synthesis [11,12,13,14,15], asymmetric catalysis [16,17,18,19,20], and radical chemistry [21,22,23,24,25], there has been a surge in the study of C-C bond-forming reactions, particularly those involving the creation of chiral carbon centers [26,27,28]. In the realm of radical reactions, extensive research has been dedicated to the discovery of innovative radical transfer reagents and the development of efficient activation methods to initiate key radical intermediates [21,22,23,24,25]. Over the past decade, a variety of novel radical transfer reagents have been synthesized and widely applied to C-C bond formation (Scheme 1) [29,30,31,32,33,34]. Examples include pyridinium salts [35,36,37,38,39,40], NHPI (N-hydroxyphthalimide) esters [41,42,43,44,45,46], 4-substituted Hantzsch esters [47,48,49,50,51], dihydroquinazolinones [52,53,54,55,56], and benzothiazolines [57,58,59,60,61,62,63,64], etc. These reagents can be categorized into two primary groups based on their activation mechanisms. The first group involves reagents that operate through a single-electron reduction activation mode. Pyridinium salts [35,36,37,38,39,40] and NHPI esters [41,42,43,44,45,46] exemplify this category, requiring the acceptance of an electron to transform into radical precursors. These precursors then release radicals, concomitantly forming stable end products such as pyridines and phthalimide-N-oxyl anions. The second group comprises reagents that function through a single-electron oxidation activation mode. In this case, 4-substituted Hantzsch esters [47,48,49,50,51], dihydroquinazolinones [52,53,54,55,56], and benzothiazolines [57,58,59,60,61,62,63,64] must undergo oxidation to release an electron, generating key radical cation intermediates. These highly reactive radical cations subsequently produce radicals, converting themselves into aromatic structures in the process. These two activation modes, single-electron reduction and single-electron oxidation activation, position them as valuable tools for a diverse array of applications in chemical synthesis.

**Scheme 2:**
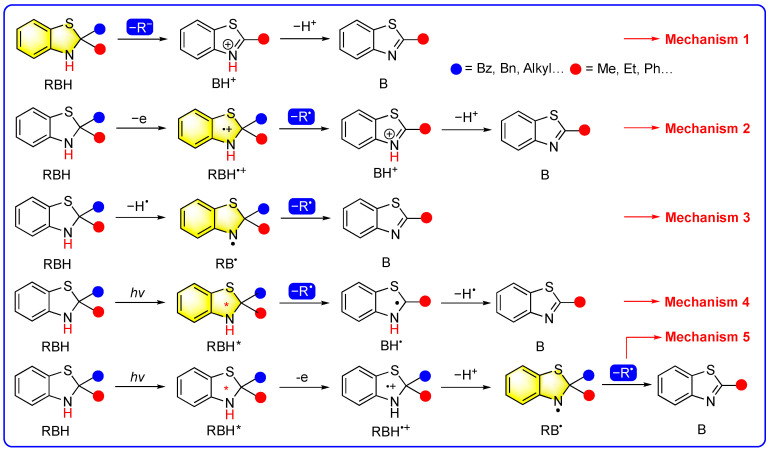
The five types of activation mechanisms of RBH acting as carbanion and radical reagents.

Traditionally employed as hydrogenation reagents [65], since 2013, benzothiazolines have emerged as versatile carbanion [57] and radical transfer reagents [58,59,60,61,62,63,64], playing a pivotal role in the construction of various carbon–carbon (C-C) bonds. They have demonstrated the ability to act as both alkyl and acyl radical reagents, with their chemical processes encompassing a diverse array of activation mechanisms, as illustrated in Scheme 2. As depicted in Scheme 2, the mechanisms can be outlined as follows: Mechanism 1 is a carbanion transfer mechanism, in which benzothiazolines (RBH) function directly as carbanion transfer reagents through a sequential R^−^ + H^+^ mechanism, resulting in the formation of aromatic benzothiazolines (B), RBH → R^−^ + H^+^ + B. Mechanism 2 shows that benzothiazolines function as radical transfer reagents through a sequential e + R^•^ + H^+^ mechanism, with RBH^•+^ serving as the radical precursors, RBH → e + R^•^ + H^+^ + B. Mechanism 3 shows that benzothiazolines operate as radical transfer reagents through a sequential H^•^ + R^•^ mechanism, with RB^•^ serving as the actual radical precursors, RBH → H^•^ + R^•^ + B. Mechanism 4 indicates that photoexcited benzothiazolines directly function as radical precursors through a sequential photoactivation + R^•^ + H^•^ mechanism, RBH → RBH* → R^•^ + H^•^ (* refers to the photoexcited state). Mechanism 5 indicates that benzothiazolines function as radical transfer reagents through a sequential photoactivation + e + H^+^ + R^•^ mechanism, with RB^•^ being the actual radical precursors, RBH → RBH* → e + H^+^ + R^•^ + B. The versatility of benzothiazolines serving as carbanion and radical transfer reagents, coupled with the variety of their activation modes and release mechanisms, underscores the potential for further innovation. Continued design and development are necessary to harness these reagents for the efficient synthesis of complex C-C bonds in organic chemistry.

Benzothiazolines indeed possess a number of distinctive advantages when utilized as carbanion and radical transfer reagents, making them highly valuable in synthetic chemistry [57,58,59,60,61,62,63,64]. Their key benefits can be highlighted as follows: (1) Ease of synthesis: Benzothiazolines can be synthetized with relative ease from readily available and cost-effective starting materials, such as ketones or aldehydes and 2-aminobenzenethiol [57,58,59,60,61,62,63,64]. This synthesis often gives high yields, making them accessible for a wide range of applications. Recently, Liao and coworkers reviewed ketone-derived pro-aromatic reagents for radical transfer reactions [66]. (2) Versatility and innovation: Benzothiazolines are versatile carbanion and radical transfer reagents. These reagents are not only versatile but also offer a broad spectrum of activation modes [57,58,59,60,61,62,63,64]. This diversity opens up significant opportunities for innovative approaches in chemical synthesis. (3) Neutral reaction conditions: Transfer reactions involving benzothiazolines typically proceed under neutral conditions, often requiring a minimal amount of catalyst or even being additive-free [57,58,59,60,61,62,63,64]. This feature simplifies reaction setups and reduces the potential for side reactions. (4) Wider substrate tolerance: The reactions are compatible with a wide array of unsaturated compounds, including but not limited to alkenes, imines, isonitriles, and heterocycles [57,58,59,60,61,62,63,64]. This tolerance allows for a high degree of functional group compatibility. (5) Efficient group transfer: Benzothiazolines serve as efficient transfer reagents for alkyl and acyl groups, enabling a variety of transformations such as hydroalkylation, hydroacylation, hydroformylation, and alkyl or acyl substitution reactions [57,58,59,60,61,62,63,64]. (6) Photoexcitability: They can be easily excited by visible light, and once excited, benzothiazolines act as excellent single-electron and radical donors [57,58,59,60,61,62,63,64]. This property makes them particularly useful in photochemical reactions, where they can initiate or participate in a variety of radical processes.

In summary, benzothiazolines’ unique combination of synthetic accessibility, versatility, operational simplicity, substrate tolerance, and photochemical reactivity positions them as powerful tools for synthetic chemists, facilitating the development of new synthetic strategies and the construction of complex molecules. From 2013 to 2024, benzothiazolines have emerged as elegant carbanion and radical transfer reagents in the construction of carbon–carbon bonds [57,58,59,60,61,62,63,64]. This review highlights their sophisticated applications, unveiling and promoting their potential in ionic and radical reactions.

## 2. Mechanism 1: Benzothiazolines Directly Acting as Carbanion Transfer Reagents

Hantzsch esters have garnered recognition for their exceptional hydrogenation property to effectively reduce unsaturated compounds [67,68,69]. Early as 2013, Tang and coworkers innovatively utilized 4-substituted Hantzsch esters as alkyl transfer reagents [57], marking a significant advancement in the field. Their work detailed the first instance of 4-substituted Hantzsch esters facilitating the alkylation of imines, employing the strong Lewis acid BF_3_·Et_2_O as a catalyst at a substantial loading of 30 mol%. Up to now, hundreds of publications have expanded the utility of 4-substituted Hantzsch esters across a spectrum of alkyl and acyl radical reactions [47,48,49,50,51].

**Scheme 3:**
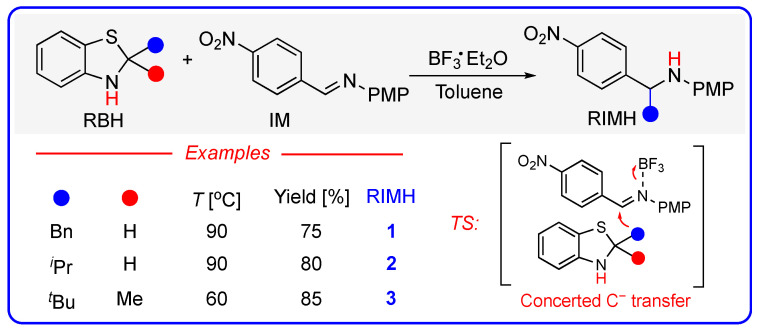
Alkyl carbanion transfer with benzothiazoles inspired by 4-substituted Hantzsch esters.

Motivated by the remarkable alkyl transfer capabilities of 4-substituted Hantzsch esters, the authors hypothesized that benzothiazoles might exhibit analogous reactivity (Scheme 3) [57]. This hypothesis was grounded in the fact that, similar to Hantzsch esters, benzothiazoles are pre-aromatic compounds with a proven track record as hydrogenation agents for the reduction of unsaturated compounds in chemical transformations. Therefore, benzothiazoles possess the potential to release alkyl groups, driven by strong aromatization forces. As expected, the alkyl group transfer (Bn, *^i^*Pr, *^t^*Bu) from benzothiazoles to imines proceeded efficiently, yielding products at high yields ranging from 75% to 85% (Scheme 3) [57]. Surprisingly, the reactivity of 2-monosubstituted-benzothiazoles (**1** and **2**) was found to be notably comparable to that of 2,2-disubstituted-benzothiazoles (**3**). These findings suggest that the presence of two alkyl substituents at the C2-position of benzothiazolines is not mandatory for benzothiazoles to function as effective alkyl transfer reagents. Moreover, the results imply that aldehydes could serve as versatile starting materials from which pro-aromatic benzothiazoles can be generated, expanding the substrate scope and synthetic potential of these transformations.

The authors carried out several experiments to elucidate the chemical reaction mechanism. Initially, TEMPO (2,2,6,6-tetramethylpiperidin-1-oxyl) was employed in an attempt to capture potential key radical intermediates [70,71,72,73]. Interestingly, the alkylation reaction proceeded well, without any loss in yield, leading to the reasonable exclusion of a radical transfer mechanism. Furthermore, if the alkylation reaction were to proceed via a radical mechanism, it would be expected that some alkyl radicals might undergo rearrangement, resulting in rearranged alkylated products. However, for the specifically designed 1-phenylpropan-2-yl-substituted alkyl transfer reagent, no rearranged alkylated product was detected. These control experiments confirm that the alkylation reactions between benzothiazoles and imines do not involve a radical mechanism. Additionally, an excess of Lewis acid catalysts had no significant impact on the alkylation reactions. Ultimately, it was demonstrated that the alkylation reactions proceed through a concerted carbanion nucleophilic addition from benzothiazoles to imines [57], catalyzed by Lewis acids.

## 3. Mechanism 2: Benzothiazolines Function as Radical Transfer Reagents Through a Sequential e + R^•^ +H^+^ Mechanism with RBH^•+^ Serving as the Radical Precursors

### 3.1. Evaluation of Benzothiazolines as Radical Transfer Reagents Through Stern–Volmer Quenching Experiments and DFT Calculations

**Scheme 4:**
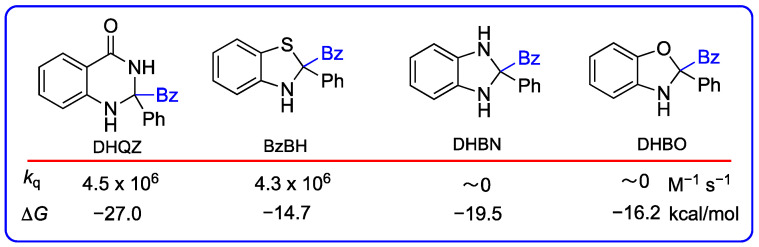
Kinetic and thermodynamic evaluations of pro-aromatic radical reagents by Stern–Volmer analysis and DFT computation.

For pre-aromatic compounds, the drive towards aromatization is a potent force for the release of groups. In 2022, the Liao group assessed the potential of key pre-aromatic compounds to serve as radical reagents in photo-promoted radical addition reactions [63]. Therefore, the single-electron oxidated reactivity of pre-aromatics to generate radical precursors, and the radical releasing ability of resulting radical cations are significant parameters to measure the potential feasibility of pre-aromatics functioning as radical transfer reagents. In Liao’s study, a variety of pre-aromatic compounds were evaluated, including dihydroquinazolinone (DHQZ), 2-benzoyl-2-phenylbenzothiazoline (BzBH), dihydrobenzimidazole (DHBN), and dihyhdrooxazole (DHBO) (Scheme 4) [63].

To measure the reactivity of the initiating radical precursors, Stern–Volmer quenching experiments with photoexcited 4CzIPN (1,2,3,5-Tetrakis(carbazol-9-yl)-4,6-dicyanobenzene) [74,75,76,77] were conducted. This approach provided valuable insights into the initial single-electron transfer steps to form the radical precursor. Moreover, to delve deeper into the thermodynamics of the radical release, the Gibbs free energies of the pre-aromatic radical cations releasing benzoyl radical were calculated using density functional theory (DFT). These calculations shed light on the energetics involved in the release of radicals from the pre-aromatic radical cations, offering a predictive tool for the potential of these compounds to act as effective radical transfer reagents.

As depicted in Scheme 4, the Stern–Volmer quenching constants (*k*_q_) were measured to be 4.5 × 10^6^ and 4.3 × 10^6^ M^−1^ S^−1^ for dihydroquinazolinone (DHQZ) and 2-benzoyl-2-phenylbenzothiazoline (BzBH), respectively. These values indicate that the SET (single-electron transfer) from these pre-aromatics to photoexcited catalysts is efficient and considerable. Moreover, the Gibbs free energies of pre-aromatic radical cation-releasing radicals (Δ*G*) are calculated to be −27.0 and −14.7 kcal/mol for DHQZ and benzothiazolines (RBH), respectively. This signifies that the radical release processes are thermodynamically feasible, driven by strong thermodynamic forces (Δ*G* << 0) [78]. By integrating the data from Stern–Volmer quenching constants (*k*_q_) and the Gibbs free energies of radical cation intermediates (Δ*G*), both DHQZ and BzBH have been identified as promising candidates for radical transfer reagents.

It is noteworthy that, although radical release from the radical cations of dihydrobenzimidazole (DHBN) and dihydrooxazole (DHBO) is thermodynamically favorable, the Stern–Volmer quenching constants (*k*_q_) are effectively zero. This suggests that the initial oxidation step of DHBN and DHBO to radical cations by photoexcited 4CzIPN is hindered, rendering them less suitable as radical transfer reagents under the given conditions. The authors speculated that employing a stronger single-electron oxidant [76,79] to activate DHBN and DHBO into radical precursors could potentially enable their use as efficient radical transfer reagents in chemical reactions. All in all, benzothiazolines (RBH) have been confirmed as promising radical transfer reagents through a combination of Stern–Volmer quenching experiments and DFT calculations. The general mechanism involves a sequential e + R^•^ +H^+^ mechanism, with RBH^•+^ serving as the radical precursors enabled by photocatalysts.

### 3.2. Hydroalkylation and Hydroacylation of Michael Acceptors via Photo-Promoted Radical Addition Reaction

In 2019, the Akiyama and Zhu groups independently investigated the application of benzothiazoles acting as radical transfer reagents under photoirradiation conditions, revealing two distinct radical transfer mechanisms [58,59]. Akiyama’s study reported a process wherein benzothiazolines act as radical transfer reagents through a sequential e + R^•^ + H^+^ mechanism, with benzothiazoline cations (RBH^•+^) being the pivotal radical precursors in this pathway. In contrast, Zhu’s research uncovered an alternative mechanism for benzothiazolines’ function as radical transfer reagents. This mechanism is characterized by a sequential H^•^ + R^•^ mechanism with RB^•^ serving as the actual radical precursors, facilitated by hydrogen abstraction reagents and the application of photoirradiation.

**Scheme 5:**
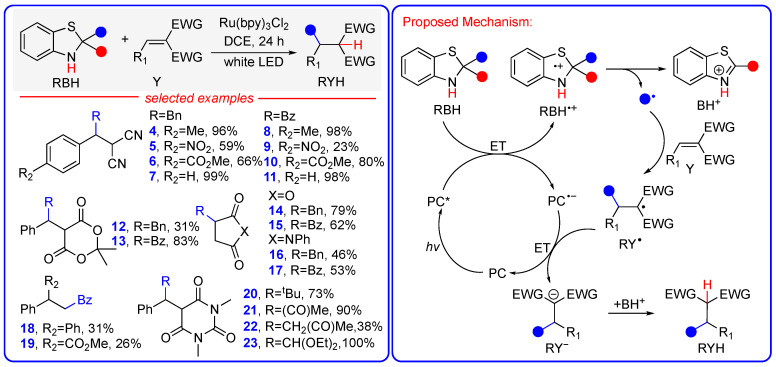
Hydroalkylation and hydroacylation of Michael acceptors via photo-promoted radical addition reaction and the corresponding proposed mechanism.

In 2019, Akiyama’s group reported a novel study on the hydroalkylation and hydroacylation of electron-deficient alkenes (Y), harnessing benzothiazolines as radical transfer reagents under photoirradiation conditions (Scheme 5) [58]. The optimized reaction conditions involved the use of DCE (1,2-dichloroethane) as the solvent, with Ru(bpy)_3_Cl_2_ (2–5 mol%) or eosin Y-2Na (2 mol%) as photocatalysts under white LED light. This system was effective for reactions with 2-benzyl-2-phenylbenzothiazoline (BnBH) and 2-benzoyl-2-phenylbenzothiazoline (BzBH), and the substrate scope of methylenemalononitriles and α,β-unsaturated carbonyl compounds was expanded.

The experimental outcomes demonstrated that hydroalkylation and hydroacylation reactions proceeded smoothly, giving products with moderate to good yields (23–99%) for aryl [4-substituted phenyl (**4**–**11**) and 2-thienyl]- or alkyl (Et, *^t^*Bu)-substituted methylenemalononitriles. With α,β-unsaturated dicarbonyl substrates, these reactions furnished moderate to excellent yields (19–91%), whereas for α,β-unsaturated monocarbonyl substrates, only hydroacylation products were obtained (23–56%), and hydroalkylation products were not detected (0%). This suggests that electron-withdrawing groups promote the radical addition process. Furthermore, a screening of various 2-substituted benzothiazolines showed that 4-substituted benzyl, *^t^*Bu (**20**), acetonyl (**22**, CH_3_COCH_2_-), phenacyl (PhCOCH_2_-), 4-sustituted benzoyl, CH_3_CO- (**21**), and ^t^BuCO- groups could efficiently transfer to electron-deficient alkenes, providing yields ranging from 18% to 76%. Notably, the diethoxymethyl group [**23**, (EtO)_2_CH-], as a formal equivalent, could be quantitatively transferred to unsaturated substrates, offering a novel and efficient method for the preparation of formylation compounds.

The hydroalkylation or hydroacylation of alkenes could serve as a gateway to more complex bioactive molecules. As a demonstration, the researchers provided an example of furan compound synthesis using the resulting hydroacylation product in a one-pot reaction. This work expands the synthetic utility of benzothiazolines and contributes to the development of photoinduced radical reactions for the synthesis of diverse molecules.

To gain the mechanistic insight, 1.3 equiv. TEMPO was introduced into the reaction as a radical scavenger [70,71,72,73]. The inhibition of both hydroalkylation and hydroacylation reactions, along with the formation of TEMPO adducts (45% for TEMPO-Bn and 56% for TEMPO-Bz), strongly suggests that these chemical reactions proceed via a radical mechanism. The generation of radicals was confirmed to be an electron-initiated process, robustly supported by the oxidation potentials of the benzothiazolines. The oxidation potentials of 2-benzyl-2-phenylbenzothiazoline (BnBH) and 2-benzoyl-2-phenylbenzothiazoline (BzBH) were determined to be 0.70 V and 0.78 V vs. SCE, respectively, indicating that they can be readily oxidized by the photoexcited photocatalysts {1.33 V vs. SCE for *E*_red_ [Ru(bpy)_3_Cl_2_*] and 1.06 vs. SCE for *E*_red_(Eosin*)}. Subsequently, the resulting benzothiazoline radical cations release radicals, simultaneously transforming into protonated aromatics (BH^+^).

Kinetic isotope effect (KIE) experiments were conducted to suggest that the electron reduction process from PC^•−^ to the radical addition intermediate (RY^•^) is likely the rate determining step for both the hydroalkylation and hydroacylation of alkenes, PC^•−^ + RY^•^ → PC + RY^−^. Based on these findings, we described the plausible mechanism in Scheme 5. Initially, a single-electron transfer (SET) occurs between benzothiazoline (RBH) and the photoexcited catalyst (PC*) to generate radical precursor RBH^•+^. Following this, a radical (R^•^) is released and trapped by alkene (Y), leading to the formation of a radical addition intermediate (RY^•^). Subsequently, a second SET event takes place from PC^•−^ to the radical addition intermediate (RY^•^), resulting in the formation of a carbanion (RY^−^). This carbanion (RY^−^) is protonated by BH^+^, yielding the desired hydroalkylation or hydroacylation product (RYH).

### 3.3. Metal- and Additive-Free Hydroacylation of Nitrones to Synthetize α-Hydroxyamino Ketones Enabled by Organic Photocatalyst

**Scheme 6:**
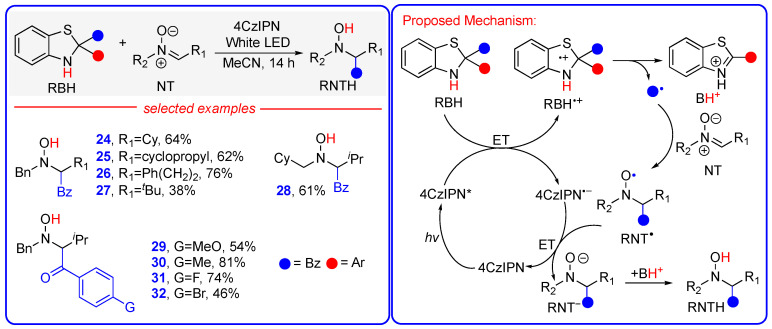
Metal- and additive-free hydroacylation of nitrones to synthetize α-hydroxyamino ketones enabled by the organic photocatalyst and the proposed mechanism.

α-Amino carbonyl compounds are recognized as significant bioactive molecules, and a variety of synthetic strategies have been elaborated to access these structures [80]. In 2023, Akiyama’s group introduced an innovative synthetic approach for the metal- and additive-free hydroacylation of nitrones (NTs) to yield α-hydroxyamino ketones (Scheme 6) [64]. This method cleverly utilizes benzothiazolines as benzoyl radical donors, enabled by an organic photocatalyst. Given the photosensitivity of the starting material, nitrone, and the susceptibility of the product, α-hydroxyamino ketone, to oxidation, the reaction conditions required meticulous optimization. Critical parameters influencing the chemical conversion and yield included the irradiation wavelength, the choice of reaction solvent, and the selection of an organic photocatalyst. For the hydrobenzoylation of N-benzyl nitrone using 2-benzoyl-2-phenylthiazoline as the acyl radical reagent, the optimal reaction conditions involved the use of acetonitrile as the solvent and 4CzIPN as the organic photocatalyst under white LED light, resulting in the α-hydroxyamino ketone in a 73% yield. When benzothiazoline was replaced with 4-benzoyl-Hantzsch ester as the benzoyl radical donor, the yield dropped significantly to 49%. This comparison underscores the superior performance of benzothiazoline as a benzoyl radical reagent, demonstrating its greater reactivity over the Hantzsch ester in this transformation.

The researchers extended their investigation to assess reaction versatility by employing a range of substituted nitrones and benzoyl-substituted benzothiazolines under standard reaction conditions (Scheme 6). The results were promising, showing that nitrones bearing cyclohexyl (**24**, 64%), cyclopropyl (**25**, 62%), phenethyl (**26**, 76%), and *^t^*Bu (**27**, 38%) groups afforded moderate and good yields (38–76%). Furthermore, benzothiazolines featuring benzoyl groups with electron-rich [methoxy (**29**) and methyl (**30**)] and electron-deficient [fluoro (**31**) and bromo (**32**)] substituents successfully participated in the reaction, affording the corresponding α-hydroxyamino ketones in another set of moderate to good yields, spanning from 46% to 81%.

Control experiments have provided compelling evidence that benzoylation reactions are entirely inhibited by the presence of TEMPO and air [70,71,72,73], confirming that the benzoyl radical derived from benzothiazolines is indeed a key intermediate in the reaction sequence. The absence of ring-opening products from the hydroacylation of cyclopropyl-substituted nitrones further supports the notion that α-amino alkyl radicals are not implicated in the reaction pathway. The proposed mechanism, as outlined in Scheme 6, begins with the oxidation of benzothiazoline by the photoexcited 4CzIPN [*E*_red_(PC*) = +1.35 V vs. SCE] through a single-electron transfer (SET) process. This event leads to the release of a benzoyl radical (R^•^) from the resultant benzothiazoline radical cation (RBH^•+^). The benzoyl radical then reacts with the nitrone (NT) to form an α-benzoylated aminoxyl radical (RNT^•^). Subsequently, a subsequent single-electron reduction and protonation mediated by 4CzIPN^•−^ and BH^+^ lead to the formation of the targeted α-hydroxyamino ketones (RNTH), with the 4CzIPN catalyst being regenerated in the process.

In conclusion, Akiyama’s group has skillfully developed a practical approach for synthesizing α-hydroxyamino ketones under metal- and additive-free conditions, harnessing the power of organic photocatalysis [64]. This method not only simplifies the synthetic route but also aligns with green chemistry principles by minimizing the use of toxic metals and additional reagents, thereby contributing to more sustainable synthetic strategies in organic chemistry.

### 3.4. Photo- and Additive-Free Hydroalkylation and Hydroacylation of Electron-Deficient Alkenes Under Thermal Condition

**Scheme 7:**
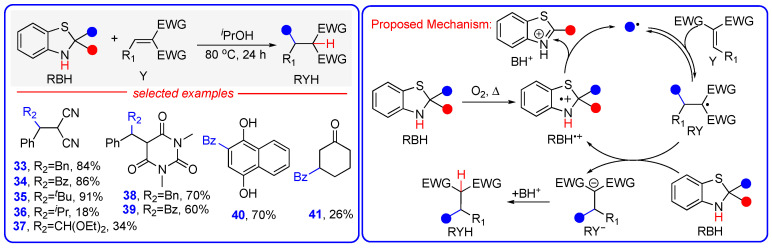
Hydroalkylation and hydroacylation of Michael acceptors under thermal conditions and the corresponding mechanism.

Traditionally, radical reactions have relied on stoichiometric amounts of organometallic reagents or catalytic amounts of photocatalysts to generate key radical species, which can be detrimental in large-scale production due to cost and toxicity concerns. However, in 2020, Akiyama’s group made a significant advancement by developing a photo- and additive-free approach for the hydroalkylation and hydroacylation of electron-deficient alkenes (Scheme 7) [60]. This innovative method utilizes benzothiazolines as radical donors under thermal conditions, eliminating the need for external irradiation or additives.

The standard reactions were optimally conducted in *^i^*PrOH for hydroalkylation and DCE for hydroacylation at 80 °C (Scheme 7). The substrate scope was extensively screened for both alkyl and acyl transfers. Notably, phenyl-substituted methylenemalononitriles (**33**–**37**) and barbituric acid derivatives (**38**–**39**) underwent efficient benzylation and benzoylation reactions, yielding products with moderate to good yields (55–98%). However, with *^t^*Bu-substituted methylenemalononitrile, the yields were somewhat lower for both benzylation (59%) and benzoylation (26%), indicating a structure-dependent reactivity. Interestingly, the benzylation of α,β-unsaturated cyclohexanone (**41**) was not observed, while the corresponding benzoylation yielded a moderate 26%. This suggests that electron-withdrawing groups facilitate the radical addition process. Furthermore, the benzothiazolines with a range of alkyl and acyl groups were investigated, and it was found that four different alkyl groups [including 4-Me-benzyl, *^t^*Bu (**35**), *^i^*Pr (**36**), and diethoxymethyl (**37**)] and three acyl groups (including 4-Me-benzoyl, 4-Br-benzoyl, and acetyl) smoothly transferred to electron-deficient alkenes, affording the desired products in yields ranging from 18% to 91%. The pivaloyl group, however, could not be transferred due to the decarbonylation of the pivaloyl radical (*^t^*BuCO^•^) to form *^t^*Bu^•^. Overall, the hydroalkylation and hydroacylation of alkenes (Y) mediated by benzothiazolines as radical transfer reagents under thermal conditions exhibit a comparable substrate scope and reactivity to those reactions conducted under photoirradiation. This indicates that the benzothiazolines are robust reagents capable of facilitating radical reactions efficiently, regardless of whether they are activated by light or heat. The thermal method offers a promising alternative that simplifies the reaction setup, potentially leading to more sustainable and practical synthetic processes.

Intuitively, the reaction was suspected to involve a mechanism where single-electron transfer (SET) occurs from benzothiazoline (RBH) to the electron-deficient alkene, followed by radical release from RBH^•+^ or a direct transfer of the alkyl or acyl group from benzothiazoline (RBH) to the electron-deficient alkene under thermal conditions. To elucidate the precise mechanism, the researchers conducted a series of experiments. The presence of TEMPO as a radical scavenger resulted in the formation of TEMPO-R adducts [70,71,72,73], and the complete inhibition of alkylation and acylation reactions of alkenes, confirming that the reaction proceeds via a radical mechanism.

The researchers also investigated the temperature dependence of the reaction yield. Notably, they found that raising the temperature from 80 °C to 100 °C (reflux) in an *^i^*PrOH solution led to a complete inhibition of the radical transfer reaction with absence of product. Similarly, the yield for the benzylation of phenyl-substituted methylenemalononitrile dropped significantly from 84% to 24% when the reaction was conducted at 80 °C in degassed *^i^*PrOH solution. Furthermore, the presence of an excess amount of O_2_ at 80 °C in an *^i^*PrOH solution caused the yield to decrease from 84% (**33**) to 50% for the same reaction. These findings suggest that the reaction only requires a certain amount of O_2_ to initiate the radical chain reaction.

The researchers proposed a plausible mechanism, as depicted in Scheme 7. Under thermal conditions, SET occurs from benzothiazoline (RBH) to O_2_, generating RBH^•+^. The subsequent release of a radical (R^•^) from RBH^•+^ is captured by the alkene (Y) to form a radical addition intermediate (RY^•^). Another SET then takes place from benzothiazoline (RBH) to the radical addition intermediate (RY^•^), producing a carbanion intermediate (RY^−^). Finally, the forming carbanion intermediate (RY^−^) is protonated by BH^+^ to yield the desired hydroalkylation or hydroacylation product (RYH). It is evident from this mechanism that a certain amount of O_2_ acts as a single-electron oxidant to generate a small amount of radical precursor RBH^•+^, after which O_2_ does not participate in the subsequent radical chain cycle.

### 3.5. Enantioselective Radical Addition of Imines Driven by the Photoexcitation of a Chiral Acid Catalyst−Imine Complex

**Scheme 8:**
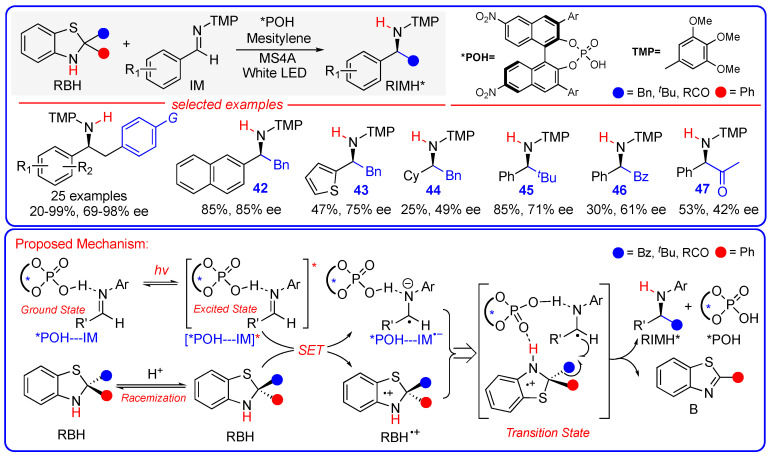
Generality of substrates and benzothiazolines and the proposed mechanism for the enantioselective radical addition of imines driven by the photoexcitation of a chiral acid catalyst−imine complex.

Chiral amines are prevalent in nature and pharmaceuticals [81,82,83], with many possessing biological activity. As a result, numerous research groups have been dedicated to developing innovative synthetic methods to access chiral amines, including ionic and radical addition reactions [81,82,83]. Recently, enantioselective radical addition reactions have garnered significant interest, particularly those driven by photocatalysis. In 2017, Melchiorre’s group reported the enantioselective alkylation of enals through the photoexcitation of iminium ions (Scheme 8), where the photoexcited iminium ions served as very strong single-electron oxidants [*E*_red_ = +2.3 V vs. SCE] [84]. Building on this work, in 2022, Akiyama’s team introduced the first example of an enantioselective radical addition to imines, powered by the photoexcitation of a chiral acid catalyst–imine complex [62].

During the experimental optimization phase, a detailed investigation was conducted on the enantioselective radical addition of N-3,4,5-trimethoxyphenyl (TMP)-substituted aldimine, utilizing 2-benzyl-2-phenylbenzothiazoline (BnBH) as a benzyl radical donor. The researchers identified that the nitro groups of chiral phosphoric acid, the TMP group of the aldimine, the presence of photoirradiation, and the use of MS4A (4A molecular sieves) were pivotal to both the reaction’s success and its enantioselectivity. When 4-benzyl-Hantzsch ester was employed in place of benzothiazoline under the optimized conditions, it yielded significantly inferior results, with a lower yield of 15% and an ee (enantiomeric excess) value of 65%. Most notably, the addition reaction was completely suppressed in the presence of air, underscoring the delicate nature of the addition process and the importance of a controlled reaction environment.

Under the optimized conditions, the researchers explored the scope of aldimines in the enantioselective radical addition reaction (Scheme 8). Initially, they examined the influence of various substituents on the aryl groups of aldimines. A range of aromatic aldimines, featuring electron-rich and electron-deficient substituents at the meta, ortho, or para positions, were found to yield amines with moderate to high yields (20–99%) and with enantioselectivities ranging from 70% to 98%. Notably, 1 and 2-naphthyl aldimines (**42**) achieved high yields (71% and 85%) and moderate enantioselectivities (86% and 85%). The 2-thienyl aldimine (**43**) provided a chiral amine with a moderate yield (47%) and with good enantioselectivity (75%). However, aliphatic imines, such as cyclohexyl aldimine (**44**), showed lower reactivity, with a yield of 25% and an enantioselectivity of 49%.

Furthermore, the researchers investigated the transfer of alkyl and acyl radicals from benzothiazolines. In terms of alkyl group transfer, para-methyl- and para-fluoro-benzyl groups reacted efficiently, yielding products with high yields (97–99%) and enantioselectivities (82–95%). The transfer involving the *^t^*Bu group (**45**) also proceeded well, affording chiral amines with moderate yields (85%) and enantioselectivities (71%). Regarding acyl group transfer, benzoyl (**46**) and acetyl (**47**) groups were introduced, resulting in products with more modest yields (30% and 53%) and moderate enantioselectivities (61% and 42%). In summary, 31 examples exhibited extensive reactivities (25–99%) and enantioselectivities (42–98%).

To unravel the underlying mechanism, a series of deliberate experiments were executed. (1) The reaction was completely inhibited by the presence of TEMPO, a radical scavenger, yielding no product and instead forming a detectable amount of TEMPO-R adducts (21%) [70,71,72,73]. This observation confirms the involvement of radical species in the reaction. (2) The quantum yield (Φ) [85] of the reaction was measured to be 0.0057, suggesting that the process is not a radical chain reaction but rather a single-event photochemical transformation. (3) The absence of radical dimer indicates that free radicals are not participating in the reaction. Instead, direct radical transfer is proposed to occur during a transient state (TS). (4) The reaction of phenyl aldimine with an excess of 2-benzyl-2-phenylbenzothiazoline (BnBH) led to the quantitative formation of product. This observation implies that benzothiazoline undergoes racemization under the optimized conditions, a hypothesis supported by the racemization reaction of deuterated benzothiazoline. (5) The formation of a complex between aldimine and chiral phosphoric acid, facilitated by hydrogen-bonding interactions, was evidenced by UV-vis spectroscopy. The photoexcited complex, which possesses strong single-electron oxidizing character (*E*_red_ was determined as +1.58 V vs. SCE), is quenched by benzothiazoline (*E*_ox_ = +0.70 V vs. SCE), driven by a substantial thermodynamic force (−20.3 kcal/mol). (6) A kinetic isotope effect (KIE) experiment using deuterated phenyl aldimine yielded a negative KIE value (*k*_H_/*k*_D_ = 0.85), indicating that the radical addition step is the rate-determining step of the reaction sequence.

A combination of the aforementioned findings, a plausible mechanism has been proposed and shown in Scheme 8. The reaction commences with the formation of a chiral acid-imine complex (*POH-IM), which is subsequently excited by visible light. The benzothiazoline, with its appropriate oxidation potential (*E*_ox_ = +0.70 V vs. SCE), is strategically positioned to undergo single-electron oxidation by the photoexcited acid-imine complex (*E*_red_{[*POH-IM]*} = +1.58 V vs. SCE) [62]. This process generates a benzothiazoline radical cation (RBH^•+^) serving as a radical precursor, and an acid–imine radical anion {[*POH-IM]^•−^}. During the transient state (TS), a radical is directly transferred from the matched benzothiazoline radical cation (RBH^•+^) to the chiral acid–imine radical anion {[*POH-IM]^•−^}, resulting in the formation of the desired chiral amine (RIMH). Concurrently, the chiral catalyst (*POH) is regenerated, setting the stage for further catalytic cycles. It is important to note that the racemization of benzothiazoline is facilitated by the presence of an acid, which is followed by the oxidation of the matched configuration by the photoexcited chiral acid–imine complex. In brief, Akiyama reported a novel enantioselective radical addition of imines driven by the photoexcited chiral acid catalyst−imine complex. The photoexcited acid–imine complex, owing to its extremely strong single-electron oxidative property, is expected to apply to more extensive photoreactions in the future.

## 4. Mechanism 3: Benzothiazolines Function as Radical Transfer Reagents Through a Sequential H^•^ + R^•^ Mechanism with RB^•^ Serving as the Actual Radical Precursors

### Acyl Radical Alkylation, Alkenylation, and Alkynylation Driven by Visible Light

**Scheme 9:**
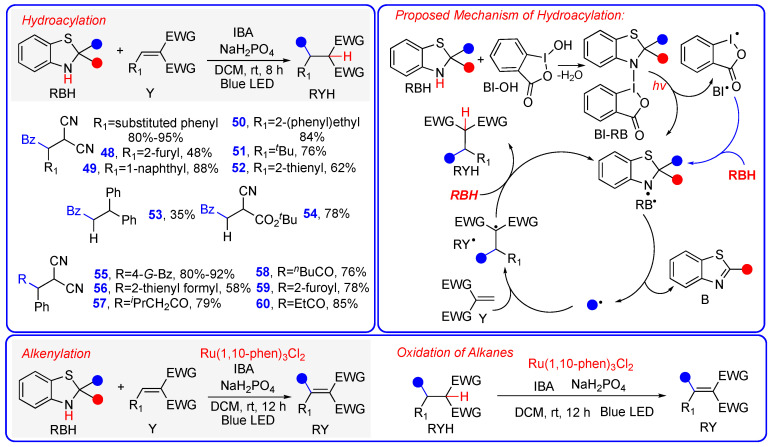
Hydroacylation of Michael acceptors and formal acyl radical alkenylation reactions under visible light-driven conditions, along with the corresponding proposed mechanism of hydroacylation of Michael acceptors.

In 2019, Zhu’s group described visible light-promoted C-C bond homolysis for acyl radical generation from benzothiazolines to achieve alkylation, alkenylation, and alkynylation reactions (Scheme 9) [59]. The researchers meticulously optimized the radical alkylation reactions of 1,1-dicyano-2-phenylethylene, utilizing 2-benzoyl-2-phenylbenzothiazoline (BzBH) as the acyl group donor. Within the reaction framework, BI-OH (hydroxybenziodoxolone) [86,87,88] was employed as either a hydrogen atom abstraction reagent or a catalyst to form radical precursors. A variety of organometallic and organic photosensitizers, varying quantities of BI-OH (from 0.3 to 2.0 equivalents), an array of bases (including NaH_2_PO_4_, Na_2_HPO_4_, Na_3_PO_4_, Na_2_CO_3_, NaOAc and KH_2_PO_4_), and several solvents (such as DCM, DCE, EtOAc, and dioxane) were systematically screened. It was determined that photosensitizers were not necessary, and the BI-OH amount could be reduced to 30 mol%. The optimized reaction conditions yielded a remarkable 92% when conducted in DCM, using 30 mol% BI-OH as a hydrogen atom abstraction catalyst at room temperature under the illumination of a blue LED.

Armed with the optimized reaction conditions, the researchers delved into exploring the substrate scope (Scheme 9). For benzothiazolines (RBH), both electron-deficient and electron-rich substituents on the phenyl ring of the benzoyl moiety were well tolerated, yielding the desired products with impressive yields ranging from 80% to 90%. The introduction of heterocycle acyl groups (**56** and **59**) led to a slight dip in yields, garnering results between 58% and 78%. Alkyl acyl groups (**57**, **58**, and **60**), on the other hand, reacted efficiently, affording yields in the range of 76% to 85%. When it came to electron-deficient alkenes, methylenemalononitriles (Y) adorned with various substituted phenyl groups engaged in the reaction seamlessly, delivering hydroacylation products with exceptional yields spanning from 80% to 95%. Methylenemalononitriles harboring heterocycles [2-furyl (**48**) and 2-thienyl (**52**)], alkyl groups [*^t^*Bu (**51**), PhCH_2_CH_2_ (**50**)], and a naphthyl group [49] also participated successfully in the reaction, yielding products in moderate to excellent yields ranging from 48% to 88%. Interestingly, the replacement of a cyano group with an ester group (**54**) resulted in a noticeable decrease in yield, from 92% to 78%. Furthermore, in contrast to phenyl-substituted methylenemalononitriles, the hydrobenzoylation yield for methylenemalononitrile (**53**) plummeted dramatically, from 92% to a mere 35%. These findings underscore the sensitivity of the reaction to the electronic nature and the substituent patterns of the substrates.

The hydroacylation reaction was fully prohibited upon the addition of TEMPO, implying that a radical species plays a pivotal role in the reaction [70,71,72,73]. Notably, with 30 mol% BI-OH, the reaction yielded over 80% of the desired products, indicating that BI-OH acts as a hydrogen atom abstraction catalyst to initiate the hydroacylation reactions, and that there may be concurrent reaction pathways. Together with the experimental outcomes, a plausible mechanism is presented in Scheme 9.

Initially, benzothiazoline (RBH) reacts with BI-OH to form an adduct BI-RB. Upon exposure to visible light, homolysis of the N-I bond occurs, resulting in the formation of RB^•^ and BI^•^ radicals. The acyl radical (R^•^) is then released from RB^•^ and gets trapped by methylenemalononitriles (Y), leading to the formation of a radical intermediate YR^•^. Subsequently, YR^•^ abstracts a hydrogen atom from another molecule of RBH, yielding the desired product YRH and regenerating another radical precursor RB^•^. Moreover, BI^•^ can also abstract a hydrogen atom from an RBH molecule, leading to the formation of a stable side product BIH and another radical precursor RB^•^. It is observed that the key radical precursor RB^•^ can be generated through three distinct routes: N-I homolysis from BI-RB, hydrogen atom abstraction by YR^•^ from RBH, and hydrogen atom abstraction by BI^•^ from RBH. This explains the use of 30 mol% BI-OH as a hydrogen atom abstraction catalyst in this reaction, ensuring a steady supply of the critical RB^•^ radical precursor.

Interestingly, during the optimization process for the reaction conditions, an unexpected outcome was observed. When a photosensitizer [2.0 mol% Ru(1,10-phen)_3_Cl_2_] [79], 1.2 equivalents of BI-OH, and 2.0 equivalents of NaH_2_PO_4_ were employed under blue LED irradiation in DCM at room temperature, radical alkenylation products (RY) were formed in yields ranging from 42 to 89% (Scheme 9). Typically, the yields of these radical alkenylation products (RY) were lower than those of the radical alkylation products (RYH). Preliminary mechanistic exploration discovered that radical alkylation products (RYH) can be effectively converted into radical alkenylation products (RY) under a photosensitizer and 1.2 equiv. BI-OH (Scheme 9). The reaction process was monitored by TLC (thin-layer chromatography). It was found that radical alkylation products (RYH) were rapidly accumulated within 2 h, and radical alkenylation products (RY) were formed accompanied by the consuming of RYH. These experiment results demonstrated a stepwise mechanism for the radical alkenylation process: the initial formation of radical alkylation products, which are subsequently subjected to oxidation by the photosensitizer and BI-OH, leading to the formation of the alkenylation products. This stepwise transformation highlights the sequential nature of the reaction pathway and the pivotal role of the photosensitizer and BI-OH in facilitating the oxidation step.

## 5. Mechanism 4: Photoexcited Benzothiazolines Directly Function as Radical Precursors Through a Sequential Photoactivation + R^•^ + H^•^ Mechanism

### Radical Alkynylation Using Photoexcited Benzothiazolines as Direct Radical Precursors

**Scheme 10:**
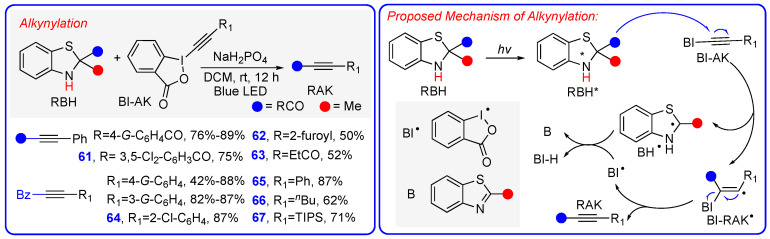
Acyl radical alkynylation reactions under visible light-driven conditions, along with the corresponding proposed mechanism.

Following the successful demonstration of acyl radical alkylation and alkenylation under visible light, Zhu’s group extended their investigation to include radical alkynylation reactions between benzothiazolines (RBH) and alkynylbenziodoxolones (BI-AK) (Scheme 10) [59]. A range of reaction parameters were meticulously optimized, including the amounts of BI-AK (1.2 to 3.0 equivalents) [89,90,91], the use of a photosensitizer, and various solvents such as DCM, DCE, EtOH, EtOAc, THF, and CH_3_CN. It was determined that the photosensitizer was not a required component of the reaction mixture. The optimal reaction conditions were established with 2.0 equivalents of NaH_2_PO_4_ in DCM at room temperature, under the irradiation of a blue LED. The scope of the reaction was then explored with a variety of benzothiazolines (RBH) and BI-AK substrates. With BI-AK, phenyl groups with both electron-withdrawing and electron-donating substituents were compatible, yielding products with moderate to excellent yields (42–88%). Alkyl [*^n^*Bu (**66**)] and silyl [TIPS: triisopropylsilyl (**67**)] groups on the acetylene moiety of BI-alkynes were also well-tolerated, affording yields of 62% and 71%, respectively. Regarding benzothiazolines (RBH), the electronic properties of the C2-acyl phenyl ring did not significantly affect the reaction outcomes, with yields ranging from 76 to 89%. However, heterocycle acyl [2-furoyl (**62**)] and alkyl acyl [EtCO (**63**)] groups showed lower reactivities with yields of 50% and 52%, respectively.

The proposed mechanism, as outlined in Scheme 10, involves the direct release of an acyl radical from the photoexcited benzothiazolines, generating a radical species (BH^•^) that reacts with BI-AK to form a radical adduct (BI-RAK^•^) [89,90,91]. The desired product is then liberated from the radical adduct (BI-RAK^•^), along with the formation of a new radical species (BI^•^). This BI^•^ radical subsequently abstracts a hydrogen atom from the residual BH^•^, leading to the formation of stable BI-H and B. It was observed that a photosensitizer was not necessary for the acyl radical alkynylation reactions, suggesting a direct role for the photoexcited benzothiazolines as radical precursors. In summary, the group achieved radical alkynylation using photoexcited benzothiazolines as direct radical precursors, with BI-AK serving as the actual source of alkynyl groups [89,90,91], marking a significant advancement in the field of radical-mediated organic synthesis.

## 6. Mechanism 5: Benzothiazolines Function as Radical Transfer Reagents Through a Sequential Photoactivation + e + H^+^ + R^•^ Mechanism with RB^•^ Serving as the Actual Radical Precursors

### Photocatalyst-Free Acylation of Quinoxaline-2-Ones and Isonitriles to Access Heterocycles Enabled by Direct Photoexcitation of Benzothiazolines

**Scheme 11:**
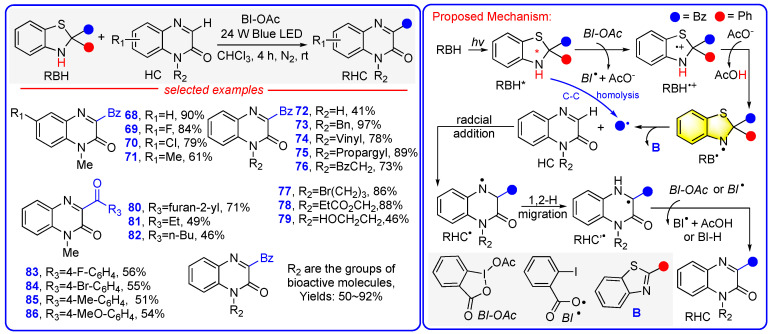
Generality of C3-acylation of quinoxaline-2-ones by benzothiazolines, along with the proposed mechanism.

**Scheme 12:**
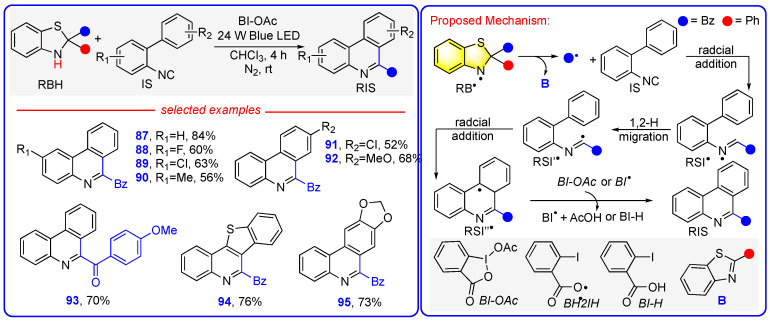
Generality of phenanthridine synthesis from isonitriles and benzothiazolines, along with the proposed mechanism.

Heterocycles are a significant class of biologically active molecules [92,93]. With the development of radical chemistry, the C_sp_^2^-H functionalization and direct construction of heterocycles have been studied intensively and applied widely especially in visible light promoted radical addition reactions filed [94,95,96]. The direct photoexcitation of substrates stands out as an economical, straightforward, and efficient strategy for radical generation [97,98]. In 2021, Xuan reported acyl radical generation from photoexcited benzothiazolines and benzothiazolines radical cations, along with their utility in accessing heterocycles (Scheme 11 and Scheme 12) [61].

At first, the researchers studied the photochemistry properties of benzothiazolines in CHCl_3_. The absorption wavelength of benzothiazolines reached 475 nm in the visible light region based on the UV-vis spectrum in CHCl_3_. Further fluorescence experiments indicated that the maximum emission wavelength (λ_max_) was 468 nm when benzothiazolines were excited at 420 nm, with an average lifetime of 1.13 ns. These findings indicated that blue LED light, ranging from 400 to 460 nm, is suitable for the photoexcitation of benzothiazolines. Building on these properties, the researchers optimized the C3-acylated reactions of N_1_-methyl-quinoxaline-2-ones with 2-benzoyl-2-phenylbenzothiazoline (BzBH) under the irradiation of blue LED. Under additive-free conditions, the yield of C3-acylated products achieved 38%, suggesting that benzothiazolines could be excited by blue LED, and the acyl group was directly released from photoexcited benzothiazolines. However, the short lifetime of photoexcited benzothiazolines limited the efficiency of acyl group transfer (yield: 38%). Therefore, supplemental acyl group release routes were needed. BI-OAc (acetoxybenzidoxole, 1.2 equiv.) [86,87,88] was added to serve as a bifunctional reagent of the electron acceptor and base. This strategic addition significantly improved the yield to 90% (**90**), demonstrating the effectiveness of BI-OAc in facilitating the acyl transfer process and enhancing the overall reaction efficiency.

Under refined reaction conditions, the researchers explored the reaction scope for both quinoxaline-2-ones and benzothiazolines (Scheme 11). For quinoxaline-2-ones, the presence of electron-withdrawing or electron-donating groups on the phenyl ring (**68**–**71**) led to the successful formation of C3-acylated products with good to excellent yields (61–84%). Moreover, quinoxaline-2-ones either unsubstituted at N1 (**72**) or substituted with various functional groups (**73**–**76**) or bioactive molecules also participated efficiently in the reaction, yielding products with moderate to excellent results (41–97%). In the case of benzothiazolines, both electron-poor and electron-rich groups on the benzoyl moiety (**83**–**86**) were well-tolerated, affording the desired products with moderate yields (51–56%). The introduction of a heterocyclic acyl group was also well-received, leading to the formation of C3-(furan-2-carbonyl)-quinoxaline-2-one (**80**) with a yield of 71%. Notably, no decarbonylation was observed for the transfer of alkyl acyl groups (**81** and **82**), which resulted in moderate yields (46% and 49%). Additionally, the team investigated the construction of phenanthridines from isonitriles and benzothiazolines (Scheme 12). They found that isonitriles bearing either electron-deficient or electron-rich groups (**87**–**95**) could smoothly engage in the reaction, yielding phenanthridines with moderate to good yields (52–84%). This part of their work further demonstrated the versatility of the photoexcited benzothiazolines in promoting radical-mediated synthesis of biologically relevant heterocycles.

Several control experiments were conducted to confirm the reaction mechanism between quinoxaline-2-ones and benzothiazolines. The chemical reactions were fully shut down when radical scavenger TEMPO was added to the reaction system. The TEMPO-R adduct was further detected by LC-MS technology. The radical trapping result indicated that a key radical specie was involved in the reaction. Moreover, the UV-vis spectrum demonstrated that electron donor–acceptor (EDA) was not formed between benzothiazolines (RBH) and BI-OAc. Considering the redox potentials, the single-electron transfer from photoexcited 2-benzoyl-2-phenylbenzothiazoline [*E*_ox_(BzBH*) = −1.68 V vs. SCE in CHCl_3_] to BI-OAc [*E*_red_(BI-OAc) = −0.64 V vs. SCE in CHCl_3_] [61] occurred efficiently driven by the strong thermodynamic driving force (−24.0 kcal/mol). As previously mentioned, under additive free condition, the direct release of the acyl group from photoexcited benzothiazolines resulted in a 30% yield.

Based on mechanistic experiments, the possible mechanism is depicted in Scheme 11. The process begins with the photoexcitation of a benzothiazoline to generate RBH* under blue LED irradiation. Following this, a single-electron transfer takes place efficiently from RBH* to BI-OAc generating RBH^•+^ and BI-OAc^•−^, RBH* + BI-OAc → RBH^•+^ + BI-OAc^•−^. Then, BI-OAc^•−^ quickly degradated into BI^•^ and AcO^−^, BI-OAc^•−^ → BI^•^ + AcO^−^. RBH^•+^ was further activated by AcO^−^ through proton transfer to form acyl radical precursor RB^•^, RBH^•+^ + AcO^−^ → RB^•^ + AcOH. RB^•^ released the key R^•^, which was trapped by quinoxaline-2-one. The generated acyl radical addition intermediate experienced 1,2-H migration, and a hydrogen atom abstraction by BI^•^ to furnish the desired C3-acylated quinoxaline-2-ones. The mechanism of phenanthridines synthesis from isonitriles and benzothiazolines was similar to this, as shown in Scheme 12. It is notable that approximately 30% acyl radicals could be afforded from photoexcited benzothiazoline (RBH*) via direct C-C bond homolysis, accompanied by the formation of the acyl radical precursor RB^•^. Therefore, the chemical reactions also experienced mechanism 4: photoexcited benzothiazolines directly functioning as radical precursors through a sequential photoactivation + R^•^ + H^•^ mechanism. All in all, Xuan reported the photocatalyst-free acylation of quinoxaline-2-ones and isonitriles to access heterocycles, enabled by a direct photoexcitation of benzothiazolines [61].

## 7. Limitations and Challenges in Benzothiazoline Chemistry

While benzothiazolines have emerged as versatile reagents in C–C bond construction, several unresolved challenges and limitations hinder their broader application in synthetic chemistry. A critical examination of these issues reveals gaps in mechanistic understanding, practical scalability, and functional group compatibility, which must be addressed to unlock the full potential of these reagents.

### 7.1. Mechanistic Ambiguities and Competing Pathways

Despite the identification of five distinct activation mechanisms, the interaction between these pathways under varying conditions remains poorly understood. For instance, the coexistence of single-electron oxidation in Mechanism 2 and hydrogen atom transfer in Mechanism 3 in photoredox systems often leads to unpredictable selectivity, especially when substrates with diverse electronic properties are employed. The lack of robust methods to suppress competing pathways limits the precision of radical transfer processes. Additionally, the role of byproducts such as aromatic benzothiazoles (B) in modulating reaction kinetics or acting as inhibitors is rarely explored. Additionally, detailed mechanistic studies using advanced spectroscopic techniques (e.g., time-resolved EPR or transient absorption spectroscopy) are needed to elucidate the lifetime and fate of intermediates like RBH^•+^ or R^•^, which could guide the design of more controlled systems.

### 7.2. Scalability and Practical Constraints

While benzothiazolines are praised for their ease of synthesis, their practical utility in large-scale reactions is hampered by several factors. Photochemical methods require specialized equipment, such as high-intensity light sources, which pose challenges in reactor design and energy efficiency. Thermal activation strategies, though additive-free, often demand elevated temperatures, raising concerns about substrate decomposition or side reactions under prolonged heating. Furthermore, the reliance on stoichiometric amounts of benzothiazolines generates stoichiometric quantities of aromatic byproducts, which are seldom recycled. Developing catalytic cycles or sustainable methods to regenerate benzothiazolines from these byproducts could enhance the atom economy and environmental viability of these reactions.

### 7.3. Functional Group Limitations and Substrate Scope

Although benzothiazolines exhibit broad substrate tolerance, their performance with sterically hindered or highly electron-rich substrates exhibits poor reactivity. For example, the hydroalkylation of α,β-unsaturated monocarbonyl compounds often fails, and the alkylation of bulky alkenes (e.g., tert-butyl-substituted alkenes) yields diminished results. Additionally, the compatibility of benzothiazolines with protic or acidic environments is underexplored, limiting their application in reactions requiring acidic additives or polar media.

### 7.4. Environmental and Economic Considerations

The synthesis of benzothiazolines from ketones/aldehydes and 2-aminobenzenethiol relies on precursors that may involve toxic or costly reagents. For instance, 2-aminobenzenethiol derivatives are not universally accessible, and their large-scale production raises sustainability concerns. Moreover, photoredox protocols often employ rare-metal catalysts (e.g., Ru or Ir complexes), which are expensive and environmentally burdensome. While organic photocatalysts like 4CzIPN offer alternatives, their long-term stability and efficiency in continuous-flow systems remain unproven.

### 7.5. Asymmetric Synthesis and Stereocontrol

The enantioselective radical additions described in Scheme 8 represent a significant advance, yet stereochemical outcomes are highly dependent on substrate-specific interactions with chiral catalysts. The limited scope of aldimines (e.g., poor reactivity with aliphatic imines) and moderate enantiomeric excess (42–98%) highlight the need for more universally applicable chiral auxiliaries or catalysts. The racemization of benzothiazolines under acidic conditions further complicates asymmetric protocols, necessitating strategies to stabilize intermediates or employ dynamic kinetic resolution.

By confronting these limitations, benzothiazoline chemistry could evolve from a laboratory tool to a cornerstone of modern synthetic strategies, bridging the gap between academic innovation and industrial applicability.

## 8. Conclusions

Benzothiazolines have emerged as transformative reagents in modern organic synthesis, enabling diverse strategies for carbon–carbon bond construction through carbanion and radical transfer pathways. This review highlights their evolution from traditional hydrogenation agents to versatile alkyl and acyl donors, classified by five distinct activation mechanisms. Key advancements include their compatibility with photoredox and thermal conditions, broad substrate tolerance, and adaptability in asymmetric catalysis. Notably, the ability to generate radicals under mild, additive-free conditions positions benzothiazolines as sustainable alternatives to conventional organometallic reagents.

The integration of benzothiazolines into photochemical workflows has unlocked novel reactivity, such as enantioselective radical additions and heterocycle synthesis, while their thermal activation bypasses the need for costly catalysts. These features align with green chemistry principles, reducing the reliance on toxic metals and energy-intensive protocols. However, challenges still persist in scalability, stereocontrol, and mechanistic predictability, which need further innovation.

Beyond methodological contributions, benzothiazoline chemistry offers broader implications for drug discovery and materials science, where efficient C–C bond formation is critical. Future efforts should prioritize mechanistic clarity, catalytic recycling of aromatic byproducts, and expansion to sterically demanding substrates. By addressing these gaps, benzothiazolines could transcend laboratory applications, becoming indispensable tools for synthesizing complex chemical molecules with precision and sustainability. It is anticipated that this review will offer exemplary applications and inspiration to synthetic chemists, contributing to the ongoing evolution of benzothiazoline utility in organic synthesis.

## Data Availability

Data are contained within the article.
